# Nipah Virus: A Neurotropic Zoonosis—From Molecular Biology to Clinical Manifestations and Translational Advances

**DOI:** 10.3390/v18090958

**Published:** 2026-09-01

**Authors:** Vignesh Mariappan, Heinz Feldmann

**Affiliations:** 1 Laboratory of Virology, Division of Intramural Research (DIR), National Institute of Allergy and Infectious Diseases (NIAID), National Institutes of Health (NIH), Rocky Mountain Laboratories (RML), Hamilton, MT 59840, USA; feldmannh@niaid.nih.gov; 2 Laboratory of Viral Zoonosis, Division of Intramural Research (DIR), National Institute of Allergy and Infectious Diseases (NIAID), National Institutes of Health (NIH), Integrated Research Facility (IRF), Frederick, MD 21702, USA

**Keywords:** Nipah virus, encephalitis, central nervous system, neuropathogenesis, therapeutics, vaccines

## Abstract

Nipah virus (NiV) is a single-stranded negative-sense RNA virus of the genus Henipavirus, family *Paramyxoviridae*. The World Health Organization has declared NiV a priority pathogen with a great public health risk due to its epidemic potential and lack of sufficient countermeasures. In humans, the virus causes Nipah viral disease (NVD), characterized by acute respiratory failure and/or encephalitis. Long-term assessments of NVD survivors have revealed persistent neurological dysfunction, including encephalopathy, cerebral atrophy, hyperintensities, ocular motor palsies, and dystonia. The entry of NiV into the central nervous system (CNS) is one of the critical and less studied aspects of its pathogenesis. NiV rapidly targets the olfactory epithelial cells in the nasal turbinate and subsequently infects neurons, allowing its spread into the CNS via axonal transport. In addition, infection of immature dendritic cells has been associated with severe CNS disease, supporting a potential role of hematogenous dissemination in neuroinvasion. Overall, understanding the molecular mechanism and the role of host factors in NiV neuropathogenesis is crucial for developing effective strategies to combat NVD. The present review delves into the neuropathogenic mechanism and immunobiology of NiV infection, as well as advancements in therapeutic modalities and vaccines against NVD.

## 1. Introduction

In 1998–1999, Malaysia experienced the first outbreak of Nipah virus (NiV), which initially caused acute respiratory disease in pigs, an intermediate/amplifying host of the virus. Humans later contracted NiV infection from infected pigs, leading to over 250 cases of febrile encephalitis among farm and abattoir workers, including 100 deaths [1,2]. This outbreak also affected Singapore, causing widespread panic and significant socioeconomic disruption in both countries. As a result, authorities culled over 1 million pigs to control the outbreak [3].

Since then, NiV infections have mainly been reported in Bangladesh and India. Starting in 2001, NiV emerged or re-emerged in several parts of Bangladesh and India, with humans mainly contracting the infection either through the ingestion of contaminated fruit or through contact with an infected person [4,5,6,7,8,9]. During these outbreaks (Table 1), indirect bat-to-human and person-to-person transmission was observed. In 2001, 75% of cases in Siliguri, India, occurred among hospital staff or visitors, indicating nosocomial transmission of NiV [10]. Additionally, an outbreak in the Philippines was associated with horse slaughter, expanding the known range of animal hosts for NiV [11]. In 2018, India witnessed a significant outbreak of Nipah virus disease (NVD) in Kerela with a case fatality rate (CFR) of 91%, in which 50% of the cases were attributed to human-to-human transmission [6,12]. However, it remains controversial whether the genetic diversity of NiV strains, a lack of healthcare or both contributed to the mortality of NVD. Since its discovery, there have been concerns about the geographic spread of NiV. Evidence of NiV infection has been detected in Pteropus bats, the natural reservoirs, which are widely distributed across southeast Asia and other countries, including Bangladesh, India, Cambodia, Thailand and Timor-Leste [13]. In contrast, serological evidence of anti-NiV or cross-reacting NiV antibodies has been detected in fruit bats from India, Bangladesh, Cambodia, China, Indonesia, Madagascar, Malaysia, New Caledonia, Papa New Guinea, Thailand and Vietnam [13,14]. Moreover, evidence of antibodies to apathogenic henipaviruses has been reported in other pteropodid bat genera, including *Eidolon* and *Rousettus*, in countries such as Madagascar and Ghana [15,16]. To date, researchers have identified two different genotypes of NiV, Malaysian (M) and Bangladesh (B), with approximately 92% genetic similarity [17]. NiV is considered a biosafety level 4 (BSL-4) pathogen due to its high mortality rate and lack of effective vaccines or therapies. It is treated as a select agent in the USA [18].

**Figure 1:**
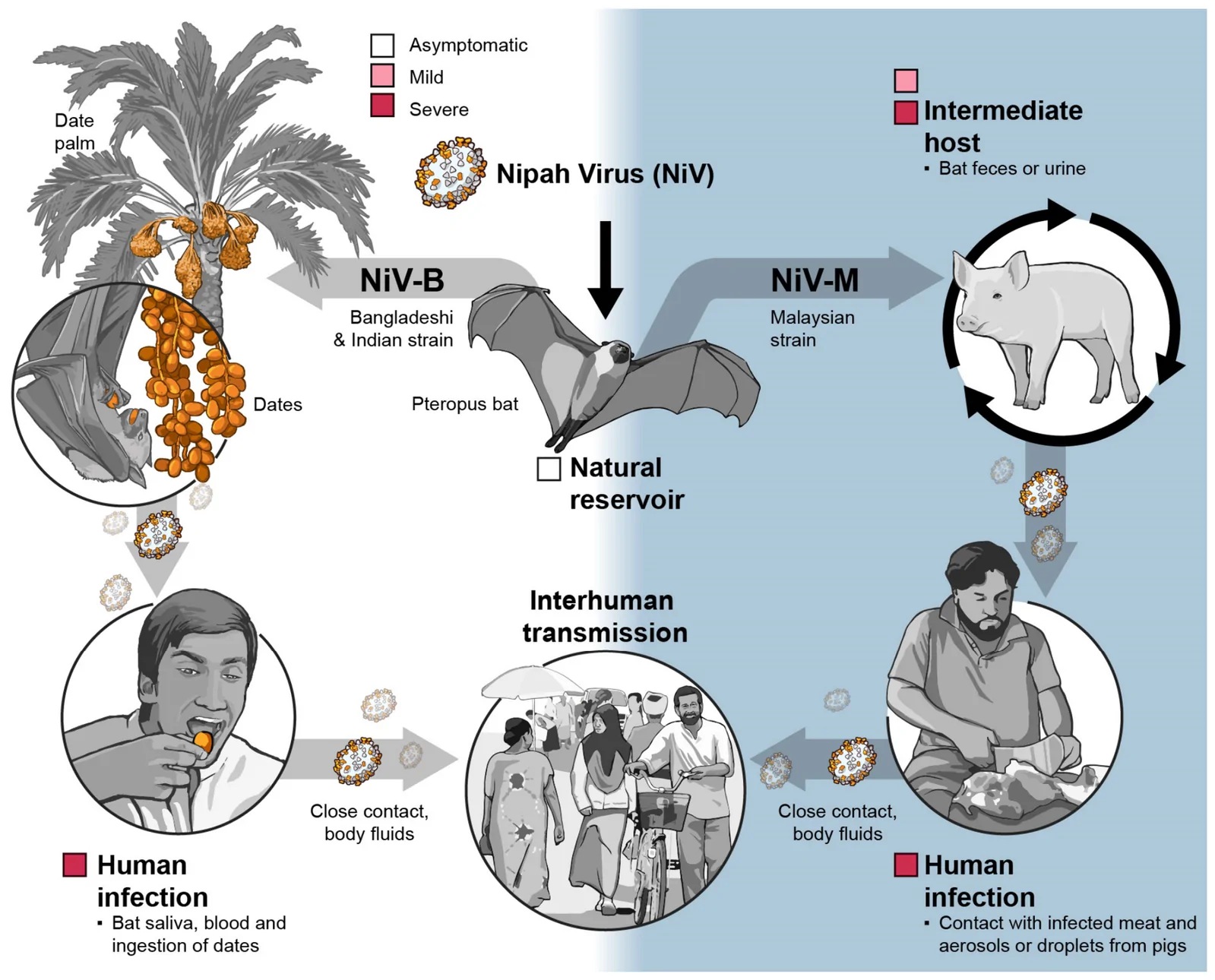
NiV transmission. *Pteropus* bats serve as a natural reservoir for NiV. In Malaysia (1999), pigs (intermediate or amplification host) were infected with NiV (NiV-M), likely through the ingestion of contaminated fruits. The virus was then transmitted to humans (end host) through handling of pigs or exposure to aerosols and droplets generated through coughing by infected pigs. Later, NiV re-emerged in different regions of Bangladesh and India (2001), leading to the transmission of NiV (NiV-B) from bats to humans via ingestion of contaminated fruit and human-to-human transmission. Human-to-human transmission could also occur through aerosols or, more likely, droplets from infected individuals with respiratory disease. Red: severe signs & symptoms; Pink: mild signs & symptoms; white: asymptomatic. Courtesy of NIAID.

The natural hosts of NiV are fruit bats of the *Pteropodidae* family, particularly species belonging to the *Pteropus* genus, such as *P. vampyrus* and *P. hypomelanus* (*Malaysian outbreaks*) and *P. medius* (formerly *P. giganteus*) in the Bangladesh and India outbreaks [38,39]. The virus can be detected in bat urine, blood, feces and saliva, with RNA detection rates of up to 3.8% in individual bats (*P. medius*). While human outbreaks are often associated with the so-called “Nipah Belt” in Bangladesh, evidence of NiV circulation has been reported in bat populations outside this region, suggesting that bat-to-human spillover events could potentially occur anywhere in Bangladesh [40]. The primary route of transmission of NiV (Figure 1) to humans seems to be related to consumption of fruit tainted with urine of NIV-infected bats [4]. Further, certain human activities, such as date palm sap harvesting and close contact with infected persons, are major drivers of bat-to-human and human-to-human transmission [41]. Therefore, systematic evolutionary studies and epidemiological investigations could provide novel insights into the dynamics of bat infections, spillover risk factors, and transmission patterns of NiV.

On the other hand, pigs played a significant role in the initial Malaysian outbreak in 1999, acting as amplifying hosts [2]. The virus is highly contagious among pigs and can also infect other domestic animals, such as horses, goats, sheep, cats, and dogs [11,42]. Therefore, characterizing the risk factors for infection in domestic animals and their potential role as amplifying hosts would provide a comprehensive way to understand the NiV ecology.

## 2. NiV Genome Organization

**Figure 2:**
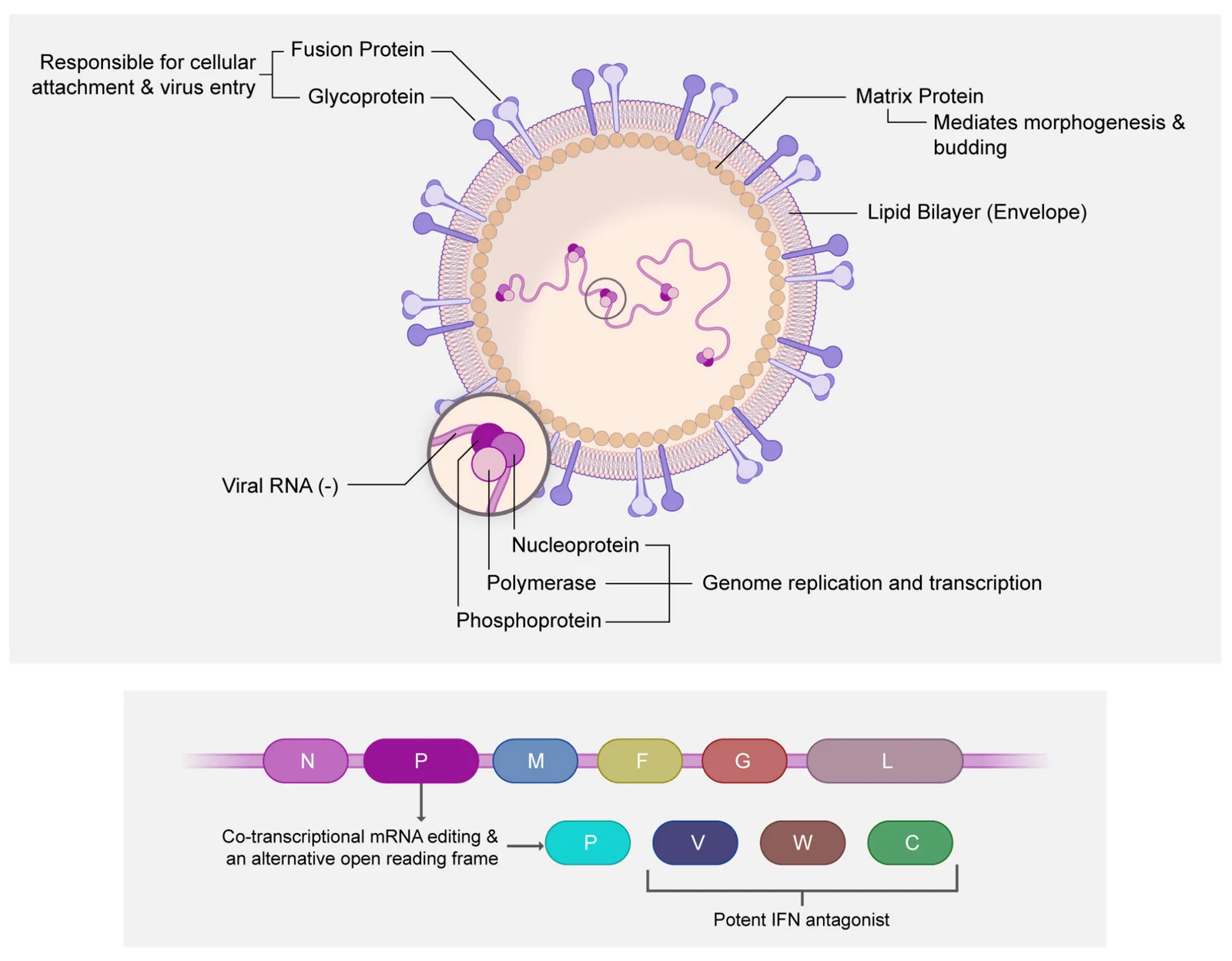
Viral genome organization and proteins of NiV. The NiV N, P, and L proteins associate with viral RNA to form a ribonucleoprotein complex that governs genome replication and transcription. This complex is encapsulated by a lipid bilayer embedded with NiV glycoproteins (F and G), which facilitate host cell attachment and viral entry. The NiV M proteins constitute the inner layer of the envelope, coordinating the budding process of progeny virions. Additionally, NiV P undergoes mRNA editing to produce the P, V, W, and C proteins, with the C protein being translated from an internal open reading frame within the gene. Courtesy of NIAID.

NiV is an enveloped, non-segmented, negative-sense, single-stranded RNA virus in the genus *Henipavirus*, family *Paramyxoviridae* [43]. The virus particles are pleomorphic, spherical to filamentous in shape, and vary in size from 40 to 1900 nm [9,29]. The genome (~18 kb) contains six genes that encode key structural proteins (Figure 2): N (nucleocapsids), P (phosphoprotein), M (matrix), F (fusion), G (glycoprotein/attachment), and L (large protein or RNA polymerase protein) [44,45].

The two glycoproteins, G and F, facilitate receptor-mediated viral entry into the host cells. Specifically, the G protein ectodomains form a homo-tetramer, serving as the viral attachment protein to bind to the host ephrin B2/B3 receptors. The F protein forms a trimer that mediates membrane fusion. Initially synthesized as an inactive precursor, the NiV F protein undergoes proteolytic cleavage by host cathepsin L and B within the endosomal recycling compartment, generating the mature fusogenic form required for viral entry [22,46,47]. The L protein serves as the catalytic core of the RNA-dependent RNA polymerase (RdRp), and the P protein acts as a cofactor, interacting with the L protein to form an RNA polymerase complex, and is crucial for both transcription and genome replication [22]. The N protein forms the helical nucleocapsid that encapsidates the viral RNA genome and interacts with the P protein during nucleocapsid formation, a crucial step in viral replication [47]. The M protein coordinates the assembly and budding of progeny viruses at the plasma membrane [47]. Furthermore, the P gene encodes three non-structural proteins (C, V, and W) that play a crucial role in suppressing interferon (IFN) production and regulates viral budding as well as release of new virions [22].

## 3. Clinical Aspects of NVD

NiV infection causes a range of clinical signs, from asymptomatic to severe. Typically, NVD manifests as flu-like symptoms, including fever, headache, cough, vomiting, myalgia, abdominal discomfort, diarrhea, gastritis, and constipation [48]. Fever is the most common symptom, followed by headaches, in 65% to 88% of NiV-infected patients. NiV infection also causes local vessel changes, such as vasculitis in small vessels in humans [49].

Despite sharing 92% nucleotide homology [17], NiV-M and NiV-B genotypes show notable differences in patient outcomes (Table 2). For example, studies in Malaysia and Singapore found that 17% to 45% of cases were asymptomatic, whereas no asymptomatic cases were reportedduring the outbreaks in Bangladesh [5,50,51]. NiV-B exhibits greater potential for human-to-human transmission compared to the Malaysian genotype, which shows less interhuman spread [52]. Consistent with this, NiV-B demonstrates higher levels of viral RNA in oral secretion in animal models [53,54,55], which may explain the increased human-to-human transmission observed with this genotype.

NiV-M infections often begin with flu-like symptoms, including fever, headache, and myalgia, followed by encephalitis with significant inflammation and necrosis, leading to severe neurological manifestations such as altered mental consciousness and seizures [56,57]. A long-term neurological study revealed that NiV-M infection can result in severe neurological sequelae in survivors, including persistent convulsions and personality changes. The CFR for NiV-M was found to be 40%, with significant morbidity among survivors [58]. MRI studies have revealed small, discrete hyperintense lesions in the subcortical and deep white matter of the brain during acute infection. These lesions were associated with inflammation, microinfarcts, necrosis and direct neuronal infection, whereas late-onset cases exhibited patchy and large confluent cortical lesions, indicating a different pathological process in relapsed compared to acute cases [58,59,60,61,62].

NiV-B infection is often associated with atypical pneumonia leading to severe pulmonary manifestation and acute respiratory distress syndrome (ARDS), with a CFR of 91% [6,9]. A spatiotemporal study of NiV-B transmission highlighted the role of respiratory involvement in person-to-person transmission [63]. A study analyzing 248 NiV-B cases in Bangladesh from 2001 to 2014 indicated that respiratory involvement significantly contributes to mortality [64]. Moreover, a 14-year surveillance study in Bangladesh noted that cough and respiratory distress were common presenting symptoms, with ARDS observed in severe cases, suggesting respiratory failure as a significant cause of mortality [65].

## 4. Neurological Aspects of NVD

Neurological manifestations are a hallmark of NiV infections in humans and typically develop during the later stages of disease. However, the mechanism underlying central nervous system (CNS) invasion remains incompletely understood.

In a hamster model, viral RNA was detected in the lungs shortly after infection, but it was not noted in the CNS until day two post-infection [66]. In addition, experimental studies in hamsters have demonstrated that olfactory sensory neurons and olfactory epithelial cells are more susceptible to NiV replication [67,68], indicating that this could be a potential route for CNS infection. Additionally, in vitro evidence indicated that hematogenous dissemination, including infection of endothelial cells (ECs) and disruption of the blood-brain barrier (BBB), may also contribute to viral entry into the CNS [69]. Furthermore, NiV-infected dendritic cells might act as Trojan horses by helping the virus cross the BBB [70]. Collectively, these findings suggest that multiple mechanisms may contribute to CNS infection, although the dominant route of neuroinvasion in humans remains unresolved.

Human autopsy studies have demonstrated widespread EC infection, characterized by systemic vasculitis and syncytia formation [62]. Specifically, parenchymal inflammation and necrosis were identified in the lungs, and NiV vasculitis was noted in the lungs, heart, kidneys, and brain [62]. Moreover, vasculitis and infected ECs were associated with platelet activation and thrombus formation, resulting in microinfarcts and distinct necrotic plaque in the gray and white matter of the brain [62]. Consistent with pathological findings, the MRI results for acute NiV infections revealed small, distinct hyperintense lesions across the brain that correlated with vascular lesions [61,71]. Viral antigens were rarely detected in glial and ependymal cells but appeared in neurons near vascular lesions [62], suggesting that vascular inflammation and BBB disruption may contribute to CNS invasion in humans.

The first reports of patients with late-onset and recurrent encephalitis were documented during the 1998–1999 Malaysian NVD outbreak [72]. Five percent of individuals in Malaysia were found to have late-onset NiV encephalitis, which is defined as the initial start of encephalitis occurring ten or more weeks after exposure to NiV. On the other hand, relapses, which are defined as the return of neurological symptoms after recovery from NiV encephalitis, were shown to occur in 9% of cases in Malaysia [73]. Both recurring and late-onset encephalitis showed similar radiological, pathological, and clinical characteristics [72]. Similarly, 18% of the 22 NiV survivors in the Bangladesh outbreak showed delayed neurological signs, such as oculomotor abnormalities, ophthalmoplegia, and cervical dystonia [58]. However, the relationship between the NiV-B virus strain and recurrent encephalitis needs further exploration. Among patients who survived acute NiV encephalitis, 66% experienced residual neurologic deficits [71]. Notably, the average time between exposure and the onset of late-onset encephalitis was found to be 8.4 months, and the average time until relapse happened was roughly 7.6 months after the primary encephalitis incident [74].

In terms of pathophysiology, late-onset or relapsed NVD showed significant viral inclusion in both neurons and neutrophils, as well as CNS involvement defined by vasculopathy, demyelination, and a massive confluent lesion [62]. Severe neuronal necrosis, gliosis, perivascular cuffing, and inflammatory infiltration are all consistent with the above findings [62]. However, several questions need to be answered, for instance: (a) Which brain cells support NiV persistence? (b) What is the role of dendritic cells as potential ‘Trojan horses’ in infection? (c) How do the neurological effects of NiV-M differ from those of NiV-B? (d) What factors contribute to recurrent or late-onset encephalitis? (e) What is the role of inflammatory mediators in the brain during infection? (f) What role do human neural stem cells play in the course of infection? (g) How do NiV proteins affect the central nervous system? And finally, is it feasible to develop an animal model for relapsed encephalitis? Addressing these gaps could significantly improve our understanding of NiV’s impact on the nervous system, potentially leading to better strategies for disease management.

## 5. Pathogenesis of NiV

**Figure 3:**
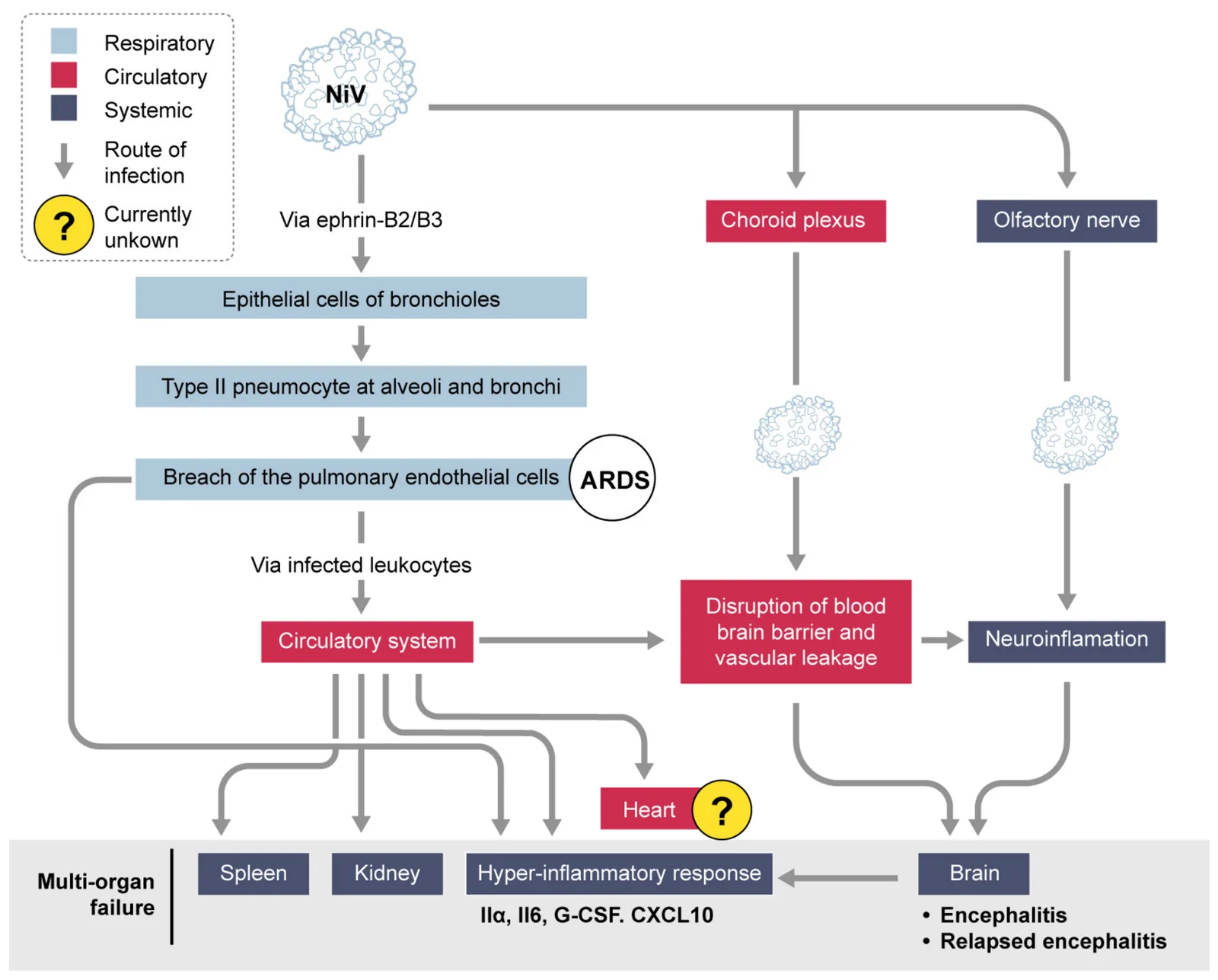
Pathogenesis of NiV infection. NiV enters the respiratory tract, likely through the oronasal route, and replicates in epithelial cells, particularly in bronchioles. The virus disseminates via the bloodstream and targets ECs, leading to vasculitis and infection of multiple organs, including the lungs, spleen and kidneys. During acute infection, viral invasion of the CNS through a disrupted BBB can result in loss of vascular integrity and neuroinflammation. After the initial acute phase, persistence of the virus within the CNS may contribute to the development of relapses or late-onset encephalitis. Courtesy of NIAID.

Initially, NiV F and G proteins facilitate the entry of the virus into cells via ephrin receptors B2 and B3 (EphrinB2/B3) [75,76]. NiV antigens can be identified in epithelial cells, specifically in type II pneumocytes and the bronchial epithelium of infected humans [77,78]. As a result, infected epithelial cells trigger the production of inflammatory cytokines in the respiratory tract, activating immune cells and eventually causing ARDS [66,77]. Studies have also observed elevated levels of inflammatory mediators, such as interleukin (IL)-1α, IL-6, IL-8, granulocyte colony-stimulating factor (G-CSF), and C-X-C motif chemokine 10 (CXCL10), from NiV-infected human respiratory epithelial cells [79,80]. In the later stage, the virus breaches the pulmonary ECs, entering the bloodstream either as free virus particles or attached to host leukocytes [81]. This allows the virus to disseminate to various organs, including the spleen, heart, kidneys, and brain, causing multi-organ dysfunction and systemic infection (Figure 3) [77,79]. However, the impact of NiV infection on cardiac function remains poorly understood.

Although the pathogenic mechanisms are broadly conserved between the two major NiV strains, differences in tissue tropism and viral replication dynamics have been proposed. NiV-B has been associated with greater respiratory involvement and efficient human-to-human transmission, whereas NiV-M outbreaks have been characterized predominantly by neurological disease and comparatively mild respiratory manifestation [57,82,83,84]. However, whether these differences in clinical and epidemiological patterns are attributable to intrinsic strain-specific properties remain unclear, and no definitive evidence has established that NiV-B and NiV-M differ fundamentally in their mechanisms of infection. Further investigations are therefore needed to determine the contribution of viral genetic variation, host factors, and virus–host interactions to the observed differences.

The virus may enter the CNS through two distinct pathways: (i) the hematogenous route, which involves the cerebrum or choroid plexus blood vessels, and (ii) the anterograde route via the olfactory nerve [85]. The hematogenous route starts with NiV entering the host through the respiratory tract, resulting in initial replication in the respiratory epithelium. Subsequently, the virus enters the bloodstream, leading to viremia. The virus then infects ECs lining the blood vessels, including those in the brain, which is a critical step in CNS entry. NiV infection can also lead to vasculitis and EC damage, compromising the integrity of the BBB [69,70,86]. Additionally, inflammatory cytokines such as IL-6, TNF-α, IL-1α, IL-1β and IFN-γ produced during infection can further contribute to BBB breakdown [77,87]. Furthermore, NiV can directly infect ECs in the CNS, leading to viral replication within these cells. This infection can result in the formation of viral inclusions, facilitating viral entry into the brain parenchyma [88]. Another potential mechanism involves leukocytes transporting NiV across the BBB, mediated by increased expression of chemokines, such as CXCL10, in the CNS, thereby contributing to viral neuroinvasion [81,89]. The hematogenous route is considered a common mechanism of CNS invasion for both NiV-B and NiV-M, although the higher respiratory viral burden associated with NiV-B may enhance systemic dissemination.

For the anterograde route, NiV initially targets and infects the olfactory epithelium in the nasal turbinate. In reference to this, an in vivo hamster model has shown that NiV can rapidly spread from the nasal epithelium along the olfactory nerve into the olfactory bulb of the brain within 3 days [68]. It also suggested that NiV uses axonal transport to move from the infected olfactory neurons through their axons. This provides a direct route for the virus to enter the CNS without needing to cross the BBB [68]. An early study involving NiV-infected pigs demonstrated that the virus could enter the CNS via cranial nerves, including the olfactory nerve, in addition to crossing the BBB directly from the bloodstream [85]. From the olfactory bulb, NiV spreads to the olfactory tubercle, and then the virus disseminates throughout the ventral cortex and areas adjacent to the lateral ventricles. Within the brain, the virus further infects neurons and glial cells, leading to vascular damage and neurological symptoms characterized by necrosis, rarefaction, spongiosis, microgliosis and encephalitis [90]. This eventually results in increased hyperinflammatory responses, which further impair vascular endothelial integrity [87]. Importantly, NiV entry into the CNS via this route coincides with the occurrence of respiratory disease, suggesting that initial CNS entry occurs simultaneously rather than sequentially through systemic virus replication. While this mechanism is well established in experimental NiV-M models, further studies are required to determine whether similar neuroinvasion dynamics occur in NiV-B and to elucidate the molecular basis for the differences in clinical manifestation observed between two genotypes. In the future, investigations should also address the primary source of inflammatory mediators in the brain, the role of infected dendritic cells, and the precise mechanism for differentiating recurrent and/or late-onset encephalitis.

## 6. Immune Evasion of NiV

The innate immune system seems to be essential for controlling NiV infection. This was supported by an early in vitro report that showed that NiV infection triggers the production of type I interferons (IFNα/β) and proinflammatory cytokines in infected ECs and airway epithelial cells [80,91,92,93]. This was highlighted in an in vivo model that demonstrated that wild-type mice are resistant to NiV, while knockout (type I IFN receptor) mice succumb to the disease [94]. This evidence suggests that the IFN response can naturally suppress NiV replication, limiting viral infection and spread.

The non-structural proteins (V, W, and C) encoded by the NiV P gene act as interferon antagonists [95]. Mechanistically, NiV V proteins can suppress the production of IFN-β by preventing the UBXN1 protein from being proteolyzed and by inhibiting retinoic acid-inducible gene I (RIG-I) and a related helicase receptor (MDA-5) [96]. This stabilizes MAVS and the ubiquitin-binding UBA domain/UBX motif, UBXN1, which leads to a dysregulated immune response, contributing to the pathophysiology of NiV and recurrent encephalitis [96,97]. In addition, it also interferes with JAK-STAT signaling by binding to STAT2, thereby blocking IFNα/β signaling transduction, while the W protein localizes to the nucleus and sequesters STAT1, blocking IFN-induced gene expression [98]. Similarly, the C protein has been shown to block TLR7/TLR9 activation by interacting with IKKα, which prevents IRF7 activation [99]. Pertaining to this, Satterfield et al. showed that deletion of the W and C genes resulted in a less severe clinical phenotype in ferrets; however, these deletions did not significantly reduce the high mortality rate and were still associated with neurological complications [93,100]. Further, the M protein interacts with and degrades the ubiquitin ligase TRIM6, which prevents the ubiquitination of IKKε, an essential process for the phosphorylation of IRF3 and STAT1 [101]. This impairs the IFN production and signaling cascades, indicating that innate immune evasion of NiV triggers a hyperinflammatory state rather than controlling viral replication. Sugai et al. (2017) outlined the crucial role of the N protein in impeding nuclear transport of STAT1, thereby suggesting a groundbreaking IFN antagonistic approach exhibited by paramyxoviruses [102]. Thus, host–virus interaction studies using an in vitro or in vivo approach could shed some light on NiV-mediated cellular interactions as well as immune evasion mechanisms.

## 7. Current Status of NiV Diagnostics

Common laboratory tests used to diagnose NiV include real-time PCR to detect the viral genome and Enzyme-Linked Immunosorbent Assay (ELISA) to detect virus-specific antibodies [103]. The traditional method used to detect NiV RNA was heminested or nested reverse transcriptase PCR (RT-PCR). This method was adopted to detect NiV with a lower limit of detection of 0.37 pg/µL of total RNA in bat urine [104]. The first real-time RT-PCR for the detection of NiV was developed based on the amplification of a conserved region of the N gene [105]. Using this technology, Zoologix created a commercial kit that can identify NiV in serum, blood, infected animal tissue, or cerebrospinal fluid [103]. More recently, three rapid molecular tests for detection of NiV were developed based on a reverse transcription recombinase-based isothermal amplification method integrated with a lateral flow assay [106]. Notably, a metagenomic approach has been developed to detect NiV in biological samples with sensitivity of 21 genomes/reaction [107].

ELISA is used to measure the antibody response. Initially, gamma-irradiated NiV antigen derived from NiV-infected cells was used to identify NiV antibodies in humans [108]. Later, NiV N protein capture ELISA based on monoclonal antibody techniques emerged to distinguish between NiV and HeV [109]. Yu et al. developed an ELISA using the recombinant NiV-N protein with 91.7% and 91.8% sensitivity and specificity, respectively [110].

Kashiwazaki et al. (2004) established a solid-phase blocking ELISA for the detection of antibodies against NiV [111]. Moreover, an indirect ELISA for IgG can detect the presence of antibodies in porcine sera against a truncated soluble HeV G or NiV G protein [112]. Interestingly, microsphere measurement has been employed to determine antibodies against a glycoprotein (NiVs G) in the serum of ruminants, including cattle, goats, and pigs [113].

Advanced technologies, including CRISPR/Cas [114], nanopore sequencing [115], plasmonic-based detection [116], and an extraction-free nucleic acid detection device [117], are being explored to detect several pathogenic viruses, including NiV.

## 8. Treatment Approaches for NiV Infection

Currently, there are no licensed approaches available to treat NiV infections. Nevertheless, monoclonal antibodies and antivirals have been studied in preclinical models and showed promising outcomes.

(a) Monoclonal Antibodies

A powerful cross-neutralizing response was shown by a human monoclonal antibody (mAb) known as m102.4 (an anti-HeV glycoprotein antibody) against both HeV and NiV by specifically targeting an epitope in the ephrin-B2 and ephirin-B3 receptor-binding sites of viral glycoprotein G [118]. In animals (ferrets and non-human primates) challenged with different henipaviruses (NiV-B, NiV-M, and HeV), this mAb exhibited protective efficacy when injected at a consistent dose [57,118,119,120]. Notably, the first-in-human dose escalation of m102.4 also demonstrated no evidence of any serious adverse events or immunogenic responses [121]. An outbreak report from India in 2018 described NiV-infected patients receiving m102.4 as postexposure prophylaxis, although efficacy data was not provided [122].

A humanized version of mouse mAb 5B3 showed cross-protectivity against NiV-M, NiV-B, and HeV in a ferret model [123]. The mouse mAbs mAb1F5 and 12B2 inhibited viral entry, while their humanized versions (h1F5 and h12B2) exhibited higher inhibitory concentration against NiV-M, NiV-B, and HeV in NHP models [124,125]. Dong et al. (2020) documented that the human mAbs HENV26 and HENV32 could block NiV-B binding to host cells via the EphB2/3 receptor [126]. Likewise, another set of anti-HeV-receptor-binding protein antibodies (HENV 103 and HENV 117) completely protected hamsters against challenge with NiV-B in combination but not individually [127].

(b) Small Molecules

Small molecules represent a promising therapeutic strategy for treating NVD. These compounds offer advantages such as oral bioavailability, ease of synthesis and the potential for broad-spectrum antiviral activity. They target various stages of the virus life cycle, including entry, replication, and host–pathogen interactions. Several candidates have demonstrated both preclinical and clinical efficacy against NVD. In animal studies, the administration of ribavirin intraperitoneally or subcutaneously delayed the onset of symptoms and death in hamsters [128,129] and African green monkeys [130] infected with NiV-M and HeV, respectively. Likewise, hamsters infected with NiV-M and treated with favipiravir survived and recovered without exhibiting any clinical signs, detectable pathology, or viral antigens upon necropsy [131]. In line with this, the nucleoside analog remdesivir was protective in African green monkeys when administered at a dose of 10 mg/kg per day starting on day 1 [132,133]. It appears that protective efficacy is dependent on early treatment, as there was only partial protection when treatment began 3 days after the initial challenge [134]. Further, ribavirin, a repurposed analog prodrug, has been used to treat NVD, showcasing its clinical effectiveness [135,136,137].

On the other hand, NiV exhibits natural resistance to the broad-spectrum allosteric paramyxovirus inhibitor GHP-88309. Recent cryo-EM analysis revealed that the resistance is due to a unique histidine residue (H1165) in the NiV L polymerase, which disrupts the inhibitor-binding pocket and prevents effective drug binding [138,139]. This structural feature distinguishes NiV from other susceptible paramyxoviruses and highlights the importance of structure-guided antiviral design for future NiV therapeutics.

## 9. Vaccine Approaches for NiV Infections

Different approaches have been tested for the development of effective NiV vaccines. The first breakthrough was achieved with a subunit vaccine approach, which used a part of the viral glycoprotein to stimulate a protective immune response. For instance, Mungall et al. observed that a soluble-glycoprotein-based subunit vaccine administered to an acutely NiV-infected feline model mediated strong antibody responses (titer ~20,000) for up to 2 months [140]. Similarly, HeVsG, a subunit glycoprotein of HeV, demonstrated exceptional outcomes in preventing disease manifestation and viral replication. Furthermore, it offered protection in a vaccinated ferret model for a period of 14 months. In contrast, pigs vaccinated with HeVsG developed cross-neutralizing antibodies against NiV but were not protected [141]. On the other hand, it is interesting to note that African green monkeys immunized with HeVsG showed protection against NiV infection, as evidenced by a high level of neutralizing and cross-reactive NiV-specific IgG antibodies [142,143]. Due to this excellent efficacy, this subunit vaccine was later developed and approved for use in horses (Equivac) in Australia [144]. Currently, the safety and immunogenicity of this vaccine are being tested in a phase I clinical trial (NCT04199169).

Various other vaccine platforms, such as viral-vectored, antiviral, chimeric, mRNA, DNA, and virus-like particle (VLP) vaccines, are under development (Table 3) for the efficient management of NiV infection. Notably, the Coalition for Epidemic Preparedness Innovation (CEPI) is engaged in producing vaccines for NiV, with four promising Nipah vaccine candidates developed by teams from academia and industry [145,146]. These are: (i) HeV-sG-V, a recombinant subunit vaccine supported by Auro Vaccines and PATH, which is administered in a two-dose regimen and has successfully completed phase I trials [147]; (ii) PHV02, a VSV-based vaccine expressing the NiV G protein supported by Public Health Vaccines, which has completed phase I trials and is progressing to phase II; (iii) a measles-virus-based vaccine supported by the University of Tokyo that is undergoing phase I and II clinical studies in Bangladesh; and (iv) the ChAdOx1-based NiV-B vaccine supported by the University of Oxford, which is in phase II testing in Bangladesh [148,149]. In addition, CEPI is also collaborating with institutions in Bangladesh and Malaysia to study NiV genetic diversity across different strains, as well as to understand immune response in NVD survivors [150,151].

## 10. Conclusions

The WHO and CEPI list NiV as a priority pathogen due to its high mortality rate, risk of pandemic transmission, and lack of licensed countermeasures. The development of different animal models has provided valuable insights into NiV pathogenesis and improved our understanding. Some of the crucial molecular mechanisms, however, need further investigations, such as: (i) the precise pathway by which NiV targets and infects the CNS; (ii) the role of host cell factors in neurological manifestations; (iii) long-term effects of NiV-mediated neurological consequences, including persistence, relapse, and late-onset disease; and (iv) the efficacy, durability, and safety of existing and upcoming vaccines, as well as therapeutic strategies against relapsed or late-onset NiV encephalitis.

Addressing these gaps requires continued refinement of animal models, including the development of models that more closely recapitulate human NVD neuropathology and immune responses. Further, studies should consider the ethical and regulatory challenges associated with the use of advanced animal models, particularly humanized models and NHPs. Additionally, parallel efforts toward the development and evaluation of safe and effective vaccines and therapeutics remain essential for improving preparedness against NiV outbreaks and reducing disease-associated mortality.

Furthermore, researchers should use novel methods that incorporate computational biology, structural biology and bioinformatics, which aid in specific and immunogenic countermeasure design. Further, pseudovirus-based systems offer an alternative cost-effective approach that could be employed in future investigations for evaluating antiviral drugs and vaccines. In the end, integration of different strategies and novel technologies will be crucial to understanding NiV pathogenesis and speeding up the process of moving vaccine candidates from the lab to real-world use.

## Figures and Tables

**Table 1:** Nipah virus outbreaks: timeline and summary.

Start Year	Location	Cases	CFR (%)	Keynote	Reference
1998	Malaysia	265	40	Pig-to-human transmission; outbreak results in large-scale pig culling	[19,20,21,22]
1999	Singapore	11	9	Occupational exposure among abattoir workers handling imported pigs form Malaysia	
2001	Siliguri, India	66	68.2	First recognized Nipah outbreak in India with substantial person-to-person/nosocomial transmission	[10]
2001	Meherpur, Bangladesh	13	69.2	First recognized Bangladesh Nipah outbreak; household clustering observed	[23]
2003	Naogaon, Bangladesh	12	66.7	Re-emergence of Nipah encephalitis; multiple household clusters identified	
2004	Manikganj & Rajbari, Bangladesh	48	75	Widespread multi-district outbreak; among the earliest evidence of recurrent Nipah spillover from fruit bats	[24]
2004	Rajbari, Manikganj, Jaipurhat, Naogang and Faridpur	22	77.3	Multi-district outbreak with 11 laboratory-confirmed cases; early evidence of widespread NiV activity	[25]
2004	Faridpur, Bangladesh	36	75	Predominantly human-to-human transmission (92%)	[26]
2005	Tangail, Bangladesh	12	91.7	First outbreak linked NiV infection to consumption of fresh raw date palm sap contaminated by fruit bats	[27]
2007	Thakurgaon District	7	42.9	First well-documented Bangladesh outbreak demonstrating sustained person-to-person transmission; six secondary cases followed close contact with the index patient	[28]
2007	Nadia, India	5	100	Intrafamilial and healthcare-associated transmission; four secondary cases followed contact with the index patient	[29]
2008	Manikganj & Rajbari, Bangladesh	10	90	Two simultaneous clusters linked to consumption of raw date palm sap; one survivor	[30]
2010	Faridpur, Bangladesh	17	88	Date-palm-sap-associated transmission and documented human-to-human transmission, including death of a healthcare worker	[31]
2014	Philippines	17	82	Horse-to-human transmission linked to horse slaughter and consumption; human-to-human spread reported	[11]
2018	Kerala, India	19	89.4	First outbreak in South India; significant nosocomial transmission	[32]
2018	Kerala, India	15	86.6	Human-to-human transmission	[33]
2021	Kerala, India	1	100	Single fatal case with limited transmission	[34]
2023	Kerala, India	6	33	Localized outbreak with evidence of nosocomial transmission	
2023	Narsingdi, Rajbari & Shariatpur, Bangladesh	11	72.7	Bat-to-human transmission through raw date palm sap consumption, no secondary spread	[35]
2024	Manikganj & Shariatpur, Bangladesh	2	100	Nipah cases linked to raw date palm sap consumption with no secondary transmission	[36]
2025	Kerala, India	4	50	First Nipah outbreak in Palakkad district	[34]
2025	Barisal, Dhaka & Rajshahi, Bangladesh	4	100	Four sporadic laboratory-confirmed fatal Nipah cases from geographically separate district; no epidemiological links were identified	[37]
2026	West Bengal, India	2	Outcome pending	Two laboratory-confirmed healthcare-worker cases; extensive contact tracing identified no further cases	[34]
2026	Rajshahi, Bangladesh	1	100	Sporadic laboratory-confirmed NiV infection associated with raw date palm date consumption; no evidence of onward transmission	[36]

**Table 2:** Key clinical differences between NiV-B and NiV-M.

Key Difference	NiV-M	NiV-B
Transmission	Pig-to-human but limited or inefficient documented human-to-human transmission	Bat-to-human and human-to-human transmission
Viral Shedding	Low in secretion of infected animals	High in oral secretion of infected animals
Asymptomatic Infections	17–45%	Infrequently reported
Case Fatality Rate	40%	68–91% (91% applies to the 2018 Kerala outbreak, not all NiV-B outbreaks)
Neurological Symptoms	Reduced consciousness, prominent brain-stem dysfunction, segmental myoclonus, areflexia, hypotonia, myoclonic jerks, abnormal doll’s eye reflex	Altered mental status, seizures, encephalitis
Respiratory Symptoms	Respiratory symptoms	Severe respiratory distress
Late-Stage Complications	Encephalitis, coma	Ophthalmoplegia, cervical dystonia, cortical lesion
Post-Recovery Complications	Neurological deficits, depression, relapses or late-onset encephalitis	Neurological deficits, relapses or late-onset encephalitis

**Table 3:** Nipah virus vaccine candidates.

	Viral Immunogen	Vaccine Name	Animal Model	Route of Vaccination	Neutralizing Antibody Titer	No. of Doses	Vaccination Scheduled	Virus Challenge	Clinical Outcome	Reference
Viral-Vectored Vaccines										
Recombinant measles virus	G	rMV-Ed-G & rMV-HL-G	Hamster	IP	Increased	2	D0 & D21	D28	Complete protection	[152]
			AGM	SC		2	D0 & D28	D42		
Recombinant Vaccinia virus	G & F	VV-NiV.G & VV-NiV.F	Hamster	SC	Increased	2	D0 & D30	3 months after booster	Complete protection	[153]
	G	MVA–NiVsG & MVA–NiV-G	Mouse	IM (humoral immunogenicity study)	Not evaluated (NiV-G-specific IgG Increased)	2	D0 & D21	-	Not evaluated (no challenge study)	[154]
				IP (T-Cell Epitope Mapping)		-	-			
Recombinant vesicular stomatitis virus	G	rVSV-ΔG-NiVBG	AGM	IM	Increased	1	D-7	D0	Complete protection	[155]
						1	D-3		Partial protection (67% survival)	
	NiV-M-G	rVSV-EBOV-GP-NiV-G	Hamster	IP	Increased	1	D-7	D0	Complete protection	[156]
						1	D-3			
						1	D-1			
						1	1 h post-infection		Partial protection (67% survival)	
						1	D1		17% survival	
						1	D3		No protection	
	G, F or G+F	VSVΔG-NiVG, VSVΔG-NiVF, or VSVΔG-NiVG/F	Hamster	IM	Higher	1	D-28	D0	Complete protection	[157]
	G or F	rVSV-ΔG-NiVBG or rVSV-ΔG-NiVBF	AGM	IM	Higher	1	D-28	D0	Complete protection	[158]
	G	rVSV-ΔG-NiVBG	AGM	IM	Higher	1	D-28	D0	Complete protection	[159]
						2	D-56 & D-28			
						1	D-365			
Recombinant rabies virus	NiV-M-G or NiV-M-F	rERAG_333E_/NiVG & rERAG_333E_/NiVF	Mouse & Pig	Oral	Increased	2	D0 & D14	-	Not evaluated (no challenge study)	[160]
	G	NIPARAB	Mouse	IM	Increased	2	D0 & D21	D49	Complete protection	[161]
ChAdOx1	G	ChAdOx1 NiV_B_	Hamster	IM	Increased	1	D-28	D0	Complete protection	[162]
						2	D-56 & D-28			
Recombinant adeno-associated virus	G, F or G+F	rAAV-NiV-G	Hamster	IM	Increased	1	D0	5 Weeks later	Complete protection	[163]
		rAAV-NiV-F			Low	1			Partial protection	
		rAAV-NiV-G + rAAV-NiV-F			Increased	1			Complete protection	
Venezuelan equine encephalitis virus replicon particles	G, F or HeV-G	VRP-NiV-G, VRP-NiV-F or VRP-HeV-G	Mouse	Footpad	Increased	2	D0 & D21	-	Not evaluated (no challenge study)	[164]
Recombinant Canarypox Virus	G, F or G+F	NiV-F (ALVAC-F), NiV-G (ALVAC-G), or both ALVAC-F + ALVAC-G	Pig	IM	Increased	2	D0 & D21	D49	Complete protection	[165]
Bovine herpesvirus	G & F	BHV-4-A-CMV-IgK-NiV-GΔTK, BHV-4-A-CMV-IgK-NiV-FΔTK or BHV-4-vectored NiV G/F	Pig	IM	Not detected/very low	2	D0 & D21	-	Not evaluated (no challenge study)	[166]
Chimeric Vaccines										
Chimeric vaccine	G & F	mRNA-LNP encoding chimeric NiV F/G vaccine	Mouse	IM	Increased	2	D0 & D21	-	Not evaluated (no challenge study)	[167]
mRNA Vaccine										
mRNA vaccine	G & F	mRNA-1215	Human	IM	-				Clinical phase I	NCT05398796
	G	HeV-sG mRNA-LNP	Hamster	IM	Increased	1	D0	D28	Complete protection	[168]
DNA Vaccines										
Pseudotype NiV	G, F or G+F	pcDNA3.1.F or pcDNA3.1.G or pcDNA3.1.F + pcDNA3.1.G	Mouse	IM	Higher	3	D0, D14 & D21	-	Not evaluated (no challenge study)	[169]
Virus-like Particles (VLPs)										
VLP	G, F & M	NiV VLP	Hamster	IM	Higher	3	D0, D21, D42	D63	Complete protection at 100 µg; partial protection at lower dose	[170]
	G, F & M		Hamster	IM	Higher	1	D-21	D0	Complete protection	[171]
						3	D0, D21 & D42	D63		
	NiV-M-G, F & M		Rabbit	IM	Increased	3	Week 0, Week 2 & Week 5	-	Not evaluated (no challenge study)	[172]
	G, F & M	HeV VLP	Mouse	IP	Not evaluated (NiV-G-specific IgG increased)	2	D0 & D21	-	Not evaluated (no challenge study)	[173]
Nanoparticle (NP)-Based Vaccine										
Ferritin-based self-assembling NP	G	NiV G ectodomain	Mouse & Hamster	IM	Higher	2	D0 & D21	Three weeks after D21	Complete protection	[174]
Self-assembled ferritin nanoparticles	G	FeNP-sG & FeNP-G_head_	Mouse & Hamster	IM	Higher	2	D0 & D21	D42		[175]

G: glycoprotein; F: fusion protein; M: matrix protein; IP: intraperitoneal; IM: intramuscular; SC: subcutaneous; AGM: African green monkey.

## Data Availability

No new data were created or analyzed in this study. Data sharing is not applicable to this article.

## Abbreviations

The following abbreviations are used in this manuscript:

NiV	Nipah Virus
NVD	Nipah Virus Disease
CNS	Central Nervous System
CFR	Case Fatality Rate
BSL-4	Biosafety Level 4
NiV-M	Nipah Virus Malaysian strain
NiV-B	Nipah Virus Bangladesh strain
N	Nucleocapsid or Nucleoprotein
P	Phosphoprotein
M	Matrix
F	Fusion
G	Glycoprotein
L	Large Protein or RNA polymerase Protein
RdRp	RNA-dependent RNA polymerase
IFN	Interferon
ARDS	Acute Respiratory Distress Syndrome
ECs	Endothelial Cells
BBB	Blood–Brain Barrier
IL	Interleukin
RIG-I	Retinoic acid-Inducible Gene-I
UBXN1	Ubiquitin-binding UBA domain/UBX motif
ELISA	Enzyme-Linked Immunosorbent Assay
RT-PCR	Reverse Transcriptase Polymerase Chain Reaction
mAb	Monoclonal Antibody
IP	Intraperitoneal
SC	Subcutaneous
IM	Intramuscular
ID	Intradermal
